# Active RNA Replication of Hepatitis C Virus Downregulates CD81 Expression

**DOI:** 10.1371/journal.pone.0054866

**Published:** 2013-01-22

**Authors:** Po-Yuan Ke, Steve S.-L. Chen

**Affiliations:** 1 Institute of Biomedical Sciences, Academia Sinica, Taipei, Taiwan, Republic of China; 2 Department of Biochemistry and Molecular Biology, Graduate Institute of Biomedical Sciences, and Molecular Medicine Research Center, College of Medicine, Chang Gung University, Taoyuan, Taiwan, Republic of China; Inserm, U1052, UMR 5286, France

## Abstract

So far how hepatitis C virus (HCV) replication modulates subsequent virus growth and propagation still remains largely unknown. Here we determine the impact of HCV replication status on the consequential virus growth by comparing normal and high levels of HCV RNA expression. We first engineered a full-length, HCV genotype 2a JFH1 genome containing a blasticidin-resistant cassette inserted at amino acid residue of 420 in nonstructural (NS) protein 5A, which allowed selection of human hepatoma Huh7 cells stably-expressing HCV. Short-term establishment of HCV stable cells attained a highly-replicating status, judged by higher expressions of viral RNA and protein as well as higher titer of viral infectivity as opposed to cells harboring the same genome without selection. Interestingly, maintenance of highly-replicating HCV stable cells led to decreased susceptibility to HCV pseudotyped particle (HCVpp) infection and downregulated cell surface level of CD81, a critical HCV entry (co)receptor. The decreased CD81 cell surface expression occurred through reduced total expression and cytoplasmic retention of CD81 within an endoplasmic reticulum -associated compartment. Moreover, productive viral RNA replication in cells harboring a JFH1 subgenomic replicon containing a similar blasticidin resistance gene cassette in NS5A and in cells robustly replicating full-length infectious genome also reduced permissiveness to HCVpp infection through decreasing the surface expression of CD81. The downregulation of CD81 surface level in HCV RNA highly-replicating cells thus interfered with reinfection and led to attenuated viral amplification. These findings together indicate that the HCV RNA replication status plays a crucial determinant in HCV growth by modulating the expression and intracellular localization of CD81.

## Introduction

**Figure 1:**
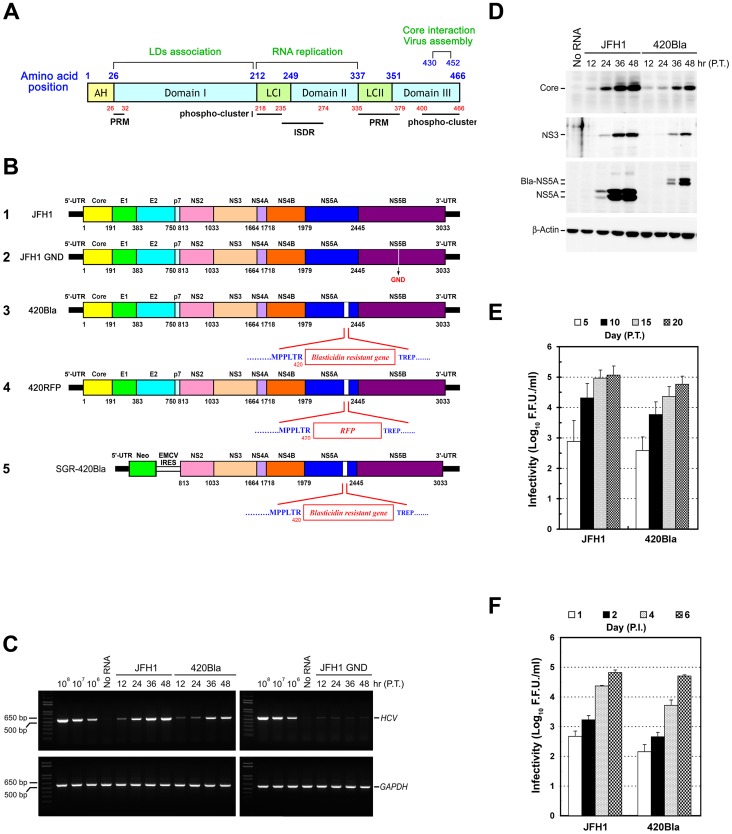
Construction and characterization of the HCV 420Bla virus. (A) Schematic representation of the domain organization of JFH1 NS5A. The locations of the N-terminal amphipathic helix (AH), three domains I, II, and III, and two low complexity (LC) regions are marked by the numbers shown on the top of the scheme. The two major phospho-clusters in LCI and Domain III represent the identified residues of hyperphosphorylation, and their corresponding regions are numbered below the scheme. The interferon sensitivity determining region (ISDR) and two proline-rich motifs (PRM) are marked by thick lines. (B) Schematic diagram of HCV RNA constructs used in this study. The relative regions of individual viral proteins encoded by JFH1 genome are represented according to their a.a. positions labeled on the translated polypeptide (scheme 1). The “GND” indicates a substitution at the 328 a.a. position of NS5B, converting the GDD motif to GND and inactivating the RNA polymerase activity of NS5B (scheme 2). The *Bla* gene was inserted at the 420 a.a. position of NS5A to yield the 420Bla genome (scheme 3). The 420RFP genome was generated by insertion of the RFP gene at 420 a.a. residue of NS5A (scheme 4). The SGR-420Bla genome (scheme 5) was constructed as described in “Materials and Methods”. (C) Huh7 cells were transfected with JFH1 or 420Bla RNAs, and the total RNAs isolated at the indicated times were analyzed by semi-quantitative RT-PCR using core- and GADPH-specific primers. P.T.: post-transfection. (D) Transfected cells were harvested at the indicated times and analyzed for expressions of core, NS3, NS5A, and β-Actin. Bla-NS5A: NS5A with a *Bla* insertion. (E) Huh7 cells were transfected with indicated viral genomes, and culture supernatants collected at different times were analyzed for the viral infectivity expressed as the foci forming unit (F.F.U.)/ml. (F) Huh7 cells were infected with the indicated viruses at an MOI of 0.01 for 12 hr, and culture supernatants harvested at the indicated times were determined for the viral infectivity. P.I.: post-infection. Data represents mean ± standard error of mean (SEM) (n = 3) (E and F).

Hepatitis C virus (HCV), a leading cause of chronic liver diseases, is an enveloped, single-stranded and positive-sense RNA virus which belongs to *Hepacivirus* genus within the family *Flaviviridae* [1]. The genome of HCV is an uncapped linear single stranded RNA molecule with a size of about 9.6 kb, which is flanked by untranslated regions (UTRs) at its 5′and 3′ ends [2]. The viral RNA encodes a single polypeptide of about 3,000 amino acids (a.a.), which is co- and post-translationally processed by a combination of cellular and viral proteases into three structural proteins (core, envelope glycoprotein E1, and E2) and seven nonstructural protein (NS) proteins (p7, NS2, NS3, NS4A, NS4B, NS5A, and NS5B) [2]. The structural proteins, core, E1 and E2, are the components of the viral particle [1], [2], whereas the NS gene products participate in the genome replication and the assembly of viral particle [2]. Among these NS proteins, NS5A is a zinc-binding protein composed of three domains separated by low complex (LC) regions LCI and LCII (Figure 1A) [3]. Domain I is responsible for the association of NS5A with lipid droplets (LDs) [3], [4]. Domain II contains the interferon sensitivity determining region (ISDR) and participates in viral RNA replication [3] whereas the C-terminus of domain III (amino acid positions 430–452) is crucial for hyperphophorylation and required for virus assembly through the interaction with core on the surface of LDs [5], [6].

Analogous to other virus infection, HCV entry into host cells relies on the specific interactions with cell surface molecules, i.e. (co)receptors that determine the binding specificity of virion and host cell tropism. Several entry (co)receptors of HCV infection, including the tetraspanin CD81, the scavenger receptor class B member I (SR-BI), and the tight junction (TJ) proteins Claudin 1 (CLDN1) and Occludin (OCLN) have been demonstrated [7]–[10]. The current model of HCV infection is that viral particles associated with lipoproteins use the glycosaminoglycans (GAGs) and the low density lipoprotein receptor (LDLR) as the initial attachment factors and target to host cell surface [11]–[13]. After binding to cell surface, SR-BI and CD81 then bind to virions with high affinity and may prime the fusogenic activity of HCV envelope glycoproteins [14]–[16]. At the postbinding step of entry into host cells, the association of CLDN1 with CD81 on the basolateral surface membrane of cells initiates the internalization process of viral particle [17], [18]. Following the internalization into cells via the pH-dependent, clathrin-mediated endocytic process, the envelope glycoproteins of virions then fuse with the endosomal membrane to release viral genome into the cytoplasm [19], [20].

Besides these entry (co)receptors, two members of CLDN family protein, CLDN6 and CLDN9, have also been shown to mediate the entry of HCV into target cells [21], [22]. In addition to be expressed in liver, CLDN6 and CLDN9 are both expressed in peripheral blood mononuclear cells which are deficient of CLDN1, suggesting the (co)receptor role of HCV infection in extrahepatic compartments [22]. Despite of these well-known HCV entry (co)factors, a functional RNAi kinase screen study has identified that epidermal growth factor receptor (EGFR) and ephrin receptor A2 (EphA2) also play its potential role in the process of HCV infection into target cells by promoting CD81-CLDN1 association and viral glycoprotein-dependent membrane fusion via their receptor tyrosine kinase (RTK) activities [23]. More recently, Sainz et al. also reported that Niemann-Pick C1-like L1 (NPC1L1), a cell surface cholesterol uptake receptor, mediates HCV entry in a cholesterol-dependent manner [24].

A recent development of an infectious system based on the HCV RNA genome of the genotype 2a JFH1, which was isolated from a Japanese patient with fulminant hepatitis C, enables the establishment of productive infection and promises the investigation of the different steps of the whole viral life cycle [12], [13], [25]. The cell culture-derived HCV (HCVcc) was shown to establish chronic persistence in vitro which triggers the coevolution between virus and host cells, thereby leading to a fluctuation of viral infectivity, a low percentage of HCV-expressing cells, and selection of cells with lower permissiveness to HCV infection [15], [16].

HCVcc infection has also been shown to downregulate the cell surface expressions of entry (co)receptors and establish superinfection exclusion to reinfection with a homologous virus in vitro [14]–[16]. In this regard, Tscherne et al. showed that HCV infection reduces CD81 via selection of a cell population expressing low levels of CD81 [15]. In contrast, Liu et al. demonstrated that the surface levels of tight junction proteins CLDN1 and OCLN1, but not CD81, are decreased to prevent superinfection in HCV-infected cells [14]. Nevertheless, how the cell surface expression of these HCV entry cofactors is reduced in HCV expressing cells is still enigmatic. Benedicto et al. showed that expression of HCV envelope glycoproteins is able to alter the localization CLDN1 and OCLN [17]. On the other hand, Farquhar et al. recently reported that internalization of HCV particle during the infection process may contribute to endocytosis of CD81 and CLDN1 from the cell surface [18].

Several HCV stable cell cultures based on a JFH1 plasmid DNA-mediated HCV RNA replication followed by antibiotic selection have been developed with an aim to robustly produce HCV [19], [20], [26]. Although the infectious virus produced from these systems mimics several aspects of the virus produced by transfection of hepatoma cells with the JFH1 RNA genome, the infectivities of viruses derived from these stable clones are unexpectedly low and cannot be promptly increased even these clones have been selected and enriched for HCV RNA- and protein-positive cells. [19], [20]. Whether the viral titers change during the establishment of these stably transfectants and what viral factors contribute to production of low-titered virus in these HCV stable clones remain to be determined.

Taking into consideration of the low titers of virus produced from these transfectants and downregulation of viral entry (co)receptors in HCV infection, we surmise that active viral RNA replication of HCV may decrease the cell surface expression of viral entry molecule, and therefore, result in a superinfection block to the subsequent viral transmission. The answers to this issue shall provide better understanding of HCV-cell interaction and viral persistence.

To address this issue, we determine in the present study how differential viral RNA replication status affects viral amplification and propagation. Toward this end, we compare viral growth and cell surface expression of CD81 in cells normally or actively expressing HCV RNA. The CD81 level on the cell surface has been shown to be a key determinant for HCV entry [27], [28]. This was achieved by respectively transfecting engineered full-length genomic and subgenomic HCV JFH1 constructs containing an insertion of a blasticidin-resistant cassette (*Bla*) in the C-terminus of NS5A, i.e., the HCV 420Bla and SGR-420Bla (Figure 1B, schemes 3 and 5, respectively), into human hepatoma Huh7 cells followed by selection with or without blasticidin.

We first found that established stable cells harboring the full-length genome at the early phase constitutively expressed high levels of viral RNA as well as proteins and high-titered virus, indicating that theses HCV stable cells attain a highly-replicating status. Nevertheless, highly active viral RNA rapidly downregulated the cell surface expression of CD81 through reducing the total CD81 protein level and cytoplasmic retention of this viral entry molecule, thereby interfering with HCV reinfection. The properties of reduced CD81 cell surface expression and resistance to HCV reinfection were also recapitulated in cells harboring the JFH1 subgenomic replicon and in cells productively expressing a chimeric strain and an adaptive mutant of JFH1 which are known to replicate more robustly than the JFH1 virus. These results together reveal, for the first time, that HCV RNA replication is crucial for viral propagation through regulating the expression and intracellular localization of CD81.

## Materials and Methods

### Cells, antibodies, and reagents

Huh7 and 293T cells were maintained in Dulbecco's modified Eagle's medium supplemented with 10% fetal bovine serum (FBS) at 37°C in an atmosphere of 5% CO_2_. In particular, nonessential amino acids were added into Huh7 growth medium at a final concentration of 1%. Anti-core antibody (Ab), anti-NS5A (9E10) Ab and anti-NS3 (CM3B6) Ab were as previously described [29]. Anti-β-actin and crystal violet were obtained from Sigma (St Louis, MO, USA). Mouse anti-CD81 (JS81), anti-CD59, anti-SR-BI, and anti-EGFR Abs were purchased from BD Biosciences (San Jose, CA, USA). Anti-CLDN1 and anti-OCLN, anti-protein disulfide isomerase (PDI), and anti-Calnexin were obtained from R & D system (Minneapolis, MN,USA) and Santa Cruz (Santa Cruz, CA, USA) respectively. The Alexa Flour-conjugated secondary Abs, 4′, 6′-diamidino-2-phenylindole (DAPI), and blasticidin were obtained from Invitrogen (Carlabad, CA, USA). Affinity-purified fluorescein isothiocyanate (FITC)-conjugated secondary antibodies, Firefly luciferase assay kit, and saponin were purchased from Kirkegaard & Perry Laboratories (Gaithersburg, MD, USA), Promega (Madison, WI, USA), and Sigma, respectively.

### Plasmid constructions

To insert a *Bla*-resistant gene into pUC-JFH1 (a construct kindly provided by T. Wakita) [12] (Figure 1B, scheme 1), a unique MluI site was created at amino acid position 420 in NS5A using oligonucleotides 5′-cctctatgccccccctcacgcgtgaggctggagatccgga-3′ (sense) and 5′-tccggatctccaggctcacgcgtgagggggggcatagagg-3′ (antisense) as a primer set by the QuikChange Site-Directed Mutagenesis kit (Stratagene, West Cedar Creek, TX, USA). The underlined sequences indicated the MluI site created by site-directed mutagenesis. The *Bla* gene was PCR amplified from the pcDNA6-TR (Invitrogen) with 5′-ccgg**acgcgt**atggccaagcctttgtct-3′ (sense) and 5′-ccgg**acgcgt**gccctcccacacataacc-3′ (antisense) as primers. The bolded and italicized sequences represent the restriction enzyme recognition sequences designed in the primers. The amplified and MluI-digested DNA fragment was inserted in the MluI site at the 420 a.a. residue, generating pJFH1420Bla (Figure 1B, scheme 3). Likewise, pJFH1420RFP was generated by insertion of the red fluorescence protein (RFP) coding sequence PCR amplified from the pDsRed-N1 plasmid (Clontech, Mountain View, CA, USA) using 5′-ccgg**acgcgt**atggcctcctccgagaac-3′ (sense) and 5′-ccgg**acgcgt**caggaacaggtggtggcg-3′ (antisense) as primers in the MluI site at the 420 residue (Figure 1B, scheme 4). To construct pSGR-420Bla (Figure 1, scheme 5), the NsiI to EcoRV fragment isolated from pJFH1-420Bla was inserted in the corresponding sites in pUC-SGR-JFH1. The full-length *Bla* gene PCR amplified with 5′- ccgg**aagctt**atggccaagcctttgtctc-3′ (sense) and 5′-ccgg**aagctt**gccctcccacacataacc (antisense) primers was subcloned in the HindIII site of pCMV22 (Sigma) to generate pCMV-FLAG-Bla. The pJ6/JFH1 and the JFH1 adaptive mutant (AM120) constructs were kindly provided by Charles Rice and Curt Hagedorn, respectively [11], [30].

### In vitro synthesis of viral genomic RNAs and generation of HCVcc

In vitro syntheses of JFH1 and its RNA genome derivatives, and production of infectious HCVcc were performed as previously described [29]. Briefly, the in vitro transcribed viral RNAs were transfected into Huh7 cells by electroporation using the Neon MicroPorator MP-100 kit (Invitrogen). Culture supernatants containing infectious virus were collected, clarified, filtrated, and stored as previously described [29].

### Infection and Titration of HCVcc

For HCVcc infection, 2×10^5^ of Huh7 cells were seeded on a 10-cm dish and 12 hr later, cells were inoculated with HCVcc at the indicated multiplicity of infection (MOI), which was supplemented with 20 mM of 4-(2-hydroxyethyl)-1-piperazineethanesulfonic acid-KOH, pH7.5, and 8 μg/ml of polybrene. Titration of viral infectivity was performed as previously described [29].

### Production and infection of HCV pseudotyped particle (HCVpp)

Pseudotyped particles encoding firefly luciferase (FLuc) and bearing HCV E1 and E2 proteins (HCVpp-FLuc) or VSV envelope glycoprotein G (VSVpp-FLuc) on the viral envelope were generated in 293T cells grown on 10-cm dishes by co-transfection of 12 μg of pTY-EF/Fluc, an HIV vector encoding a firefly luciferase reporter gene under control of the human elongation factor promoter and 12 μg of pCMVΔR8.91, a plasmid encoding HIV Gag, Pol, Rev, and Tat proteins, along with 6 μg of pcDNA3-E1E2, a construct expressing HCV E1 and E2 of the H77 strain of genotype 1a [31] or pCAGGS-E1E2-FLAG, a construct expressing E1 and E2-FLAG proteins of the JFH1 strain of genotype 2a or with 1.5 μg of pMD.G, a VSV envelope glycoprotein G-expressing plasmid using a standard calcium phosphate coprecipitation method. The culture supernatants containing pseudotyped viral particles were harvested 48 hr post-transfection, filtered through 0.45 μM filters, and stored at −80°C For infection, Huh7 cells were seeded in a 24-well plate at a density of 1×10^4^/well 18 hr prior to infection. Cells were then spin-inoculated with pseudotyped viral particles in the presence of 8 μg/ml of polybrene and cultured for 24 hr. Forty-eight hours post infection, cell lysates were determined for the firefly luciferase.

### Semi-quantitative RT-PCR analysis

To semi-quantify the intracellular amounts of HCV RNAs, total cellular RNAs were harvested using the TRIzol reagent (Invitrogen), extracted by phenol/chloroform, and then subjected to reverse transcription into cDNAs with the SuperScript III kit (Invitrogen). The cDNAs were PCR amplified for the HCV core region (nt. 365–938), the *Bla* gene, and the glyceraldehyde 3-phosphate dehydrogenase (GAPDH) gene using 5′-ccggaagcttatgagcacaaatcctaaacctcaaaga-3′ (sense) and 5′-ccgggaattcgcagcagagaccggaacggt-3′ (antisense), 5′-ccggacgcgtatggcctcctccgagaac-3′ (sense) and 5′-ccggacgcgtcaggaacaggtggtggcg-3′ (antisense), and 5′-ccacccatggcaaattccatggca-3′ (sense) and 5′-tctagacggcaggtcaggtccacc-3′ (antisense) as primer sets, respectively. Given copy numbers of in vitro transcribed JFH1 RNAs spiked with cellular RNAs were used as the standards.

### Cell viability assay

Cell viability was determined by the CellTiter 96 Non-Radioactive Cell Proliferation MTS Assay (Promega). In general, cells were first seeded in a 96-well plate at a density of 5×10^3^/well and 72 hr after inoculation, cells were washed, replenished with the fresh medium containing the MTS reagent, and cultured at 37°C for 1 hr. The number of living cells was quantified by measuring the absorbance of the reduced MTS at 490 nm.

### Immunofluorescence and confocal analysis

For titration of viral infectivity and determination of the percentage of HCV NS5A-positive cells, HCV infected cells were fixed by pre-cold methanol, permeabilized with 0.1% Triton X-100 in phosphate-buffered saline (PBS), stained with anti-NS5A Ab, and then incubated with a FITC-conjugated secondary antibody. Nuclei were labeled DAPI. For immunostaining of NS3, cells were fixed with 4% paraformaldehyde, followed by permeabilization with 0.1% Triton X-100 in PBS. Cells were then incubated with anti-NS3 Ab followed by incubation with appropriate cognate Alexa Fluor–conjugated secondary Ab. For immunostaining analysis of CD81, cells were fixed by 4% paraformaldehyde and permeabilized by 0.1% Triton X-100, followed by incubation with anti-CD81 Ab and a respective secondary Ab. The images were analyzed by laser scanning confocal microscopy; model LSM 510 (Carl Zeiss, Germany).

### Flow cytometric analysis of cell surface expression of CD81

For analysis of the cell surface expressions of (co)receptors, 2×10^6^ cells were resuspended in 100 μl fluorescence-activated cell sorting (FACS) buffer (2% FBS and 0.05% sodium azide in PBS) and incubated on ice for 4 hr with primary Ab at a dilution of 1∶100, followed by incubation with the appropriate fluorescence-conjugated secondary Ab. Then the cells were washed and fixed in 2% paraformaldehyde. The bound antibody was detected by flow cytometry. For double staining of HCV expression and surface expression of (co)receptors, the cells were first stained for (co)receptors with corresponding Abs according to the procedure described above and then permeabilized in a buffer containing PBS plus 0.5% saponin and 1% bovine serum albumin (BSA) for 30 min at room temperature. Then the cells were incubated with anti-NS5A Ab and then stained with an Alexa fluor 555-conjugated IgG2a secondary Ab. The FACS analysis was performed using the FACSCalibur flow cytometer and CellQuest software (BD Bioscience).

## Results

### The JFH1 virus with an insertion of a *Bla* gene at the residue 420 of NS5A is infectious

Previous studies have shown that chimeric constructs containing fluorochrome gene-tagged JFH1 genome and bicistronic, JFH1-based, full-length genome or subgenomic RNA containing a neomycin-resistance gene allow discrimination between the two different viral genomes in the homologous superinfection [15], [32], implying that an engineered HCV viral genome with an inserted target gene cassette can provide an alternative strategy to analyze HCV-host cell interaction. To understand how HCV interacts with cells under a more defined cell-virus context, we investigated the importance of viral replication status to subsequent viral propagation and how HCV establishes persistent infection. We first established a cell model capable of naturally or actively expressing HCV by genetically engineering a full-length JFH1 RNA genome containing a *Bla* gene inserted at the a.a. position 420 of the NS5A domain III, i.e., 420Bla genome (Figure 1B, scheme 3). Similar to JFH1 RNA, 420Bla genome produced accumulative amounts of viral RNAs as well as viral proteins core, NS3, and NS5A up to 48 hr post-transfection (Figure 1C and 1D). As expected, transfection with the full-length GND RNA (Figure 1B, scheme 2), a JFH1 genome carrying an inactivated NS5B RNA polymerase, did not replicate at all (Figure 1C). Infectious viral particles were produced in 420Bla-transfected cells in a time-dependent manner albeit with slightly slower replication kinetics than those in JFH1-transfected cells (Figure 1E). Moreover, the 420Bla virus displayed similar replication kinetics to the parental JFH1 virus when equal amounts of these viruses were inoculated into Huh7 cells (Figure 1F). These findings indicate that expression of HCV in this HCV-Bla culture system can serve as a physiological model to study the HCV replication.

### The 420Bla+ stable cells produces higher-titered infectious virus at the early stage of culture

**Figure 2:**
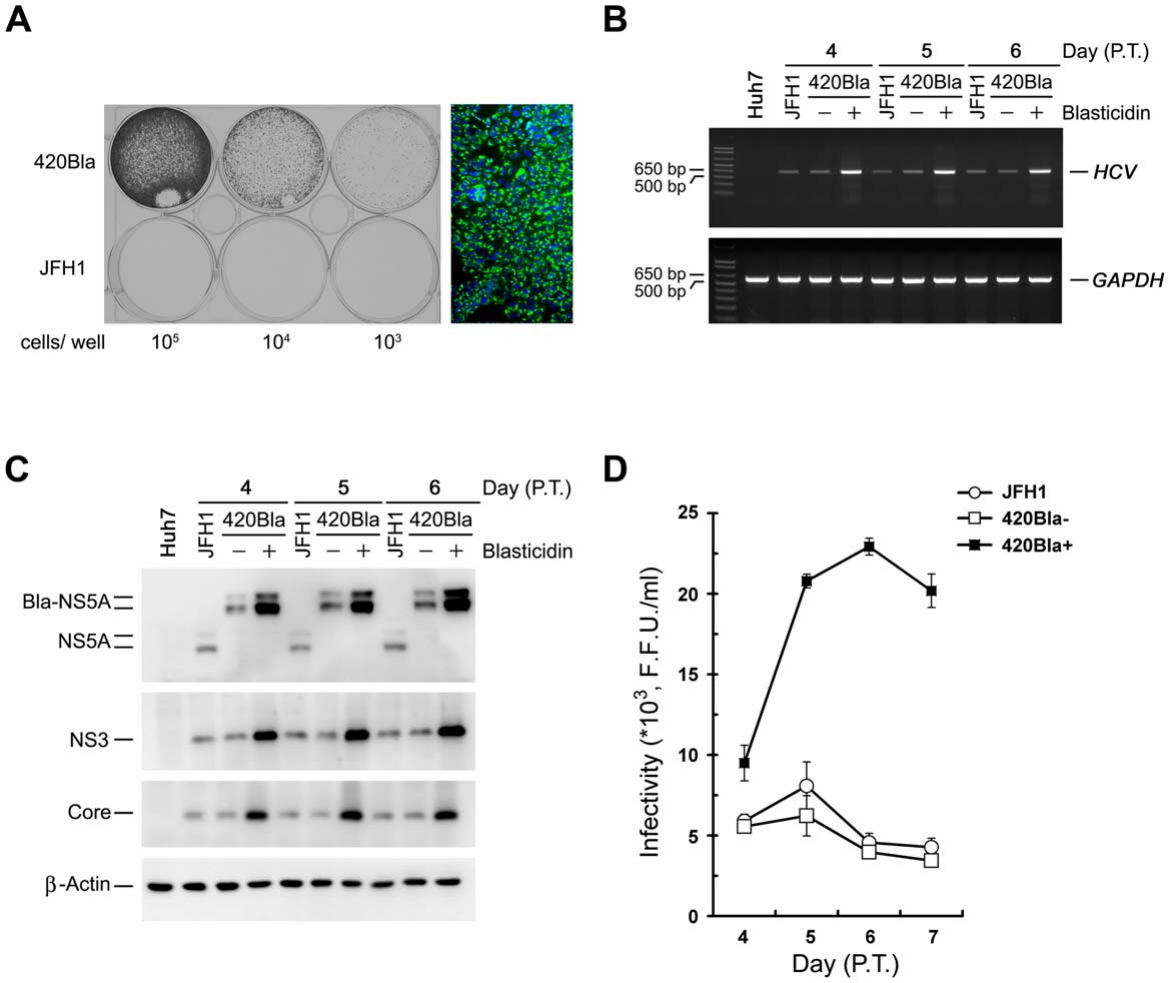
Maintenance of 420Bla+ stable cells at a productively replicating state in early stage after transfection. (A) Huh7 cells were transfected with JFH1 or 420Bla RNAs and one day after transfection, cells were seeded at the indicated densities and selected with 10 μg/ml of blasticidin for 4 days. The survival clones were visualized by crystal violet staining. The right panel represents the immunostaining of NS5A protein in blasticidin-resistant cells. Green: NS5A; Blue: nuclei stained by DAPI. (B) Huh7 cells were transfected with JFH1 or 420Bla RNAs and one day after transfection, cells were selected with or without blasticidin. Total cellular RNAs isolated at the indicated times were analyzed for the intracellular RNA amount by semi-quantitative RT-PCR. (C) Cell lysates from another set of cells as shown in (B) were harvested at indicated times and analyzed for expressions of each indicated proteins by Western blotting. (D) Culture supernatants collected from (B) were determined for the viral infectivity. Data represents mean ± SEM (n = 3) (D).

**Figure 3:**
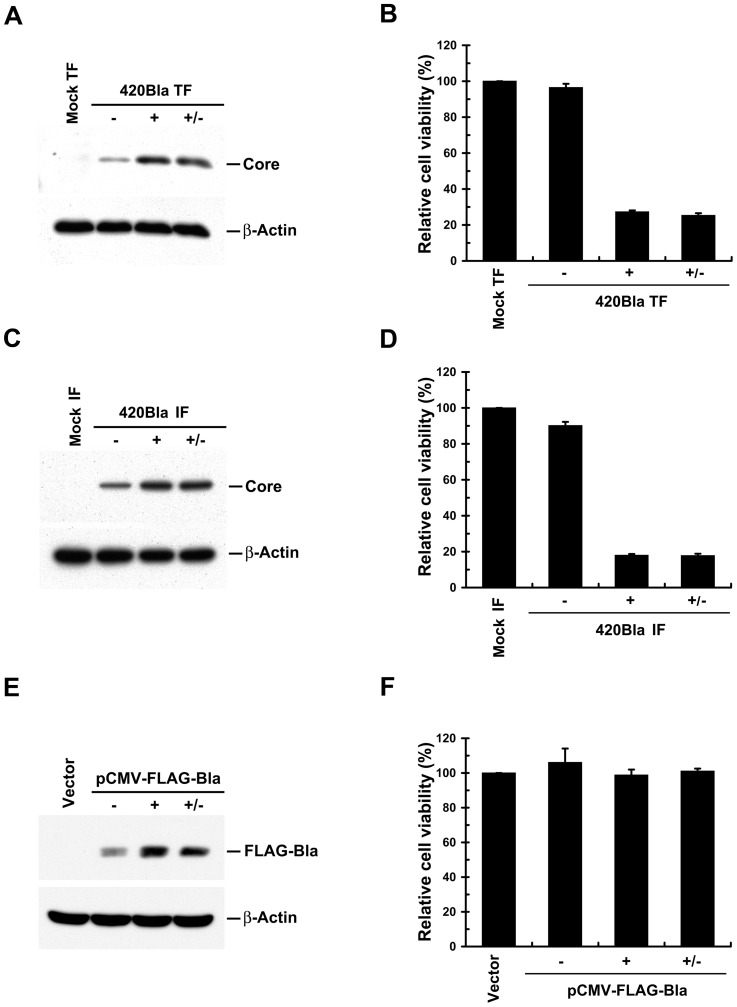
Determination of cell viability in 420Bla+ HCV stable cells. (A and B) Huh7 cells were mock transfected or transfected with 420Bla RNA. Twenty-four hours later, cells were selected with (+) or without (−) 10 μg/ml of blasticidin for 4 days. The Mock TF represents mock-transfected Huh7 cells. The “+/−” indicates the 420Bla-transfected cells selected with blasticidin for 3 days and replenished with fresh medium for 1 day. The cells were analyzed for protein expressions (A) and cell viability (B) as described in “Materials and Methods”. (C and D) Huh7 cells were mock infected or infected with 420Bla virus at an MOI of 0.01 for 24 hr. Three days later, cells were selected with (+) or without (−) blasticidin for 4 days. The “Mock IF” represents Huh7 cells without 420Bla virus infection. The “+/−” indicates the 420Bla-infected cells selected with blasticidin for 3 days and replenished with fresh medium for 24 hr. The cells were then analyzed for protein expressions (C) and cell viability (D). (E and F) Huh7 cells were transfected with an pCMV-FLAG-Bla-expressing plasmid or an empty vector. At day 2 post-transfection, cells were selected with (+) or without blasticidin for 4 days. The Vector represents Huh7 cells transfected with empty vector. The “+/−” indicates the pCMV-FLAG-Bla transfected cells selected with blasticidin for 3 days and replenished with fresh medium for 1 day. The cells were assayed for protein expression (E) and cell viability (F). Data represents mean ± SEM (n = 3).

Next, we established 420Bla stable cells by selection of 420Bla RNA-transfected cells with blasticidin for 4 days. The 420Bla RNA-transfected cells vigorously grew in the presence of blasticidin and almost all selected cells were HCV-positive (Figure 2A). Higher levels of viral RNA and proteins were detected in the 420Bla stable cells compared to those of JFH1 RNA- and 420Bla-transfected cells without selection (420Bla-) (Figure 2B and C). Also, the infectivity of virus produced from 420Bla stable cells was approximately 4-fold higher than those produced from JFH1- and 420Bla-transfected cells without selection at 5 and 6 days after transfection (Figure 2D). Moreover, 420Bla+ stable cells reduced cell growth more significantly than mock-transfected and 420Bla-transfected cells without selection (Figure 3A and B). Similarly, the cell growth was dramatically inhibited in the cells infected with 420Bla virus and selected with blasticidin whereas the 420Bla-infected cells without drug selection retained a comparable growth capacity to that of mock-infected cells (Figure 3C and D). These results are consistent with a previous report that productive replication of infectious HCV JFH1 *in vitro* triggers cytopathic effects, such as retardation of cell growth [16]. Notably, withdrawal of drug for one day from the culture media after the cells were selected with blasticidin for 3 days did not rescue the growth of 420Bla+ transfected and infected stable cells (Figure 3B and D). Nevertheless, the growth of cells harboring a CMV promoter-driven FLAG-tagged *Bla* gene (FLAG-Bla) under blasticidin selection was similar to those observed in empty vector-transfected cells and the same transfectant without drug selection (Figure 3E and F). These observations suggest that the retardation of cell growth in 420Bla+ stable cells is mainly resulted from highly replicating HCV rather than the unexpected side effect of blasticidin. Together, these results demonstrate that this strategy establishes a cell system which efficiently attains an HCV highly replicating status.

### Single clones isolated from HCV actively expressing cells show an impaired viral infectivity phenotype

**Figure 4:**
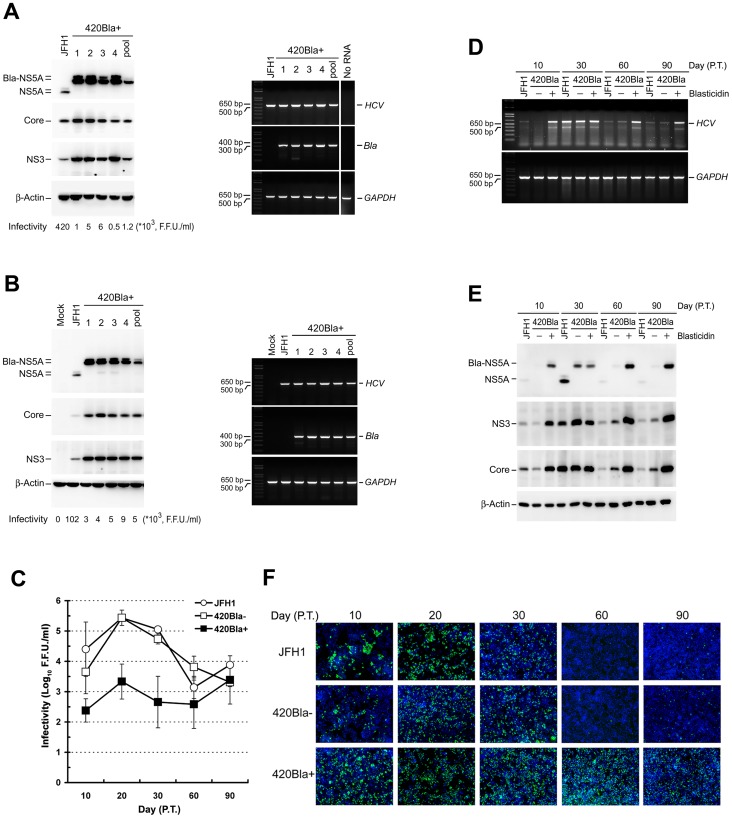
Characterization of long-term cultured 420Bla+ HCV stable cells. (A and B) Huh7 cells were respectively transfected with JFH1 and 420Bla RNAs and cultured for additional two days (A) or infected with the indicated viruses at an MOI of 0.01 for 12 hr and cultured for additional 3 days (B). Single clones from transfected or infected cells were isolated and grown in 10-cm culture dishes in the presence of 10 μg/ml of blasticidin. When reaching the confluence, the cells were harvested for analyses of the viral protein expression (left panel), extracellular viral infectivity (the numbers shown in the bottom of the left panel), and intracellular viral RNA amount (right panel). The “pool” represents the collection of all the remaining survival cells after single clone isolation whereas the “JFH1” indicates the JFH1 RNA-transfected (A) or JFH1 virus-infected (B) cells without blasticidin selection and cultured in parallel. The “No RNA” in (A) indicates the mock-transfected Huh7 cells, and the “Mock” in (B) indicates Huh7 cells without HCV infection. (C–E) Huh7 cells were transfected with JFH1- or 420Bla- RNAs and cultured for additional one day. Then one half each of transfected cells were cultured in the absence (−) or presence (+) of blasticidin. The extracellular viral infectivity (C), intracellular viral RNA amounts (D), and viral protein expressions (E) of samples harvested at the indicated times were determined. (F) JFH1, 420Bla-, and 420Bla+ cells collected at the indicated times after RNA transfection were fixed and analyzed by immunostaining of NS5A (green) and nuclei (blue). Data represents mean ± SEM (n = 3) (C).

Next, we isolated 420Bla+ single clones from transfection and infection experiments. In general, more abundant amounts of viral RNA and proteins were detected in each of the 420Bla+ transfectants, compared to those detected in JFH1 RNA-transfected cells (Figure 4A). Intriguingly, the viral infectivity of 420Bla+ selected clones was generally at least two orders of magnitude lower than that of the parental JFH1 RNA-transfected cells (Figure 4A). Similarly, all 420Bla+ clones isolated from 420Bla virus infection also expressed high levels of viral RNA and proteins, and their viral infectivity was much lower than that obtained from JFH1 virus-infected cells (Figure 4B). The JFH1 RNA-transfected Huh7 stable cells harboring a FLAG-Bla, i.e., Huh7-FLAG-Bla cells, under blasticidin selection produced infectious virus with kinetics similar to those observed in JFH1 RNA-transfected Huh7 cells and Huh7-FLAG-Bla cells without drug selection (data not shown). These results demonstrate that the lower viral infectivity of HCV-Bla stable clones cannot be attributable to the detrimental effect of blasticidin selection on viral infectivity, but rather to a deteriorating effect of highly active HCV RNA replication on viral growth.

### Active and prolonged expression of HCV fails to support production of infectious virus in the long-term culture

Next, we examined whether long-term culture of HCV transfectants can improve viral infectivity. JFH1 RNA- and 420Bla RNA-transfected cells without blasticidin selection (420Bla-) exponentially produced infectious viruses between day 10 to 20 post-transfection, with a maximum titer of about 10^5^ F.F.U./ml at day 20 after transfection; however, the viral infectivity started to fluctuate after day 20 to day 90 post-transfection (Figure 4C), which was similar to the result obtained by Zhong et al. [16]. In contrast, the viral infectivity of 420Bla+ cells was slightly increased and reached at a peak of 2.1×10^3^ F.F.U./ml at day 20 post-transfection and was not continuously increased in the long-term culture period (Figure 4C). Nevertheless, 420Bla+ cells at days 60 and 90 post-transfection still produced higher levels of RNA and proteins, as compared to JFH1- and 420Bla-tranfected cells without selection (Figure 4D and E). Also, nearly 100% of 420Bla+ stable cells expressed HCV at different times after RNA transfection whereas JFH1- and 420Bla-transfected cells without selection scarcely showed HCV-positive cells (below 5%) at day 60 and 90 post-transfection (Figure 4F). These results indicate that the 420Bla+ cells still maintain an active viral RNA replication status even at the time when low-titered virus was produced.

**Figure 5:**
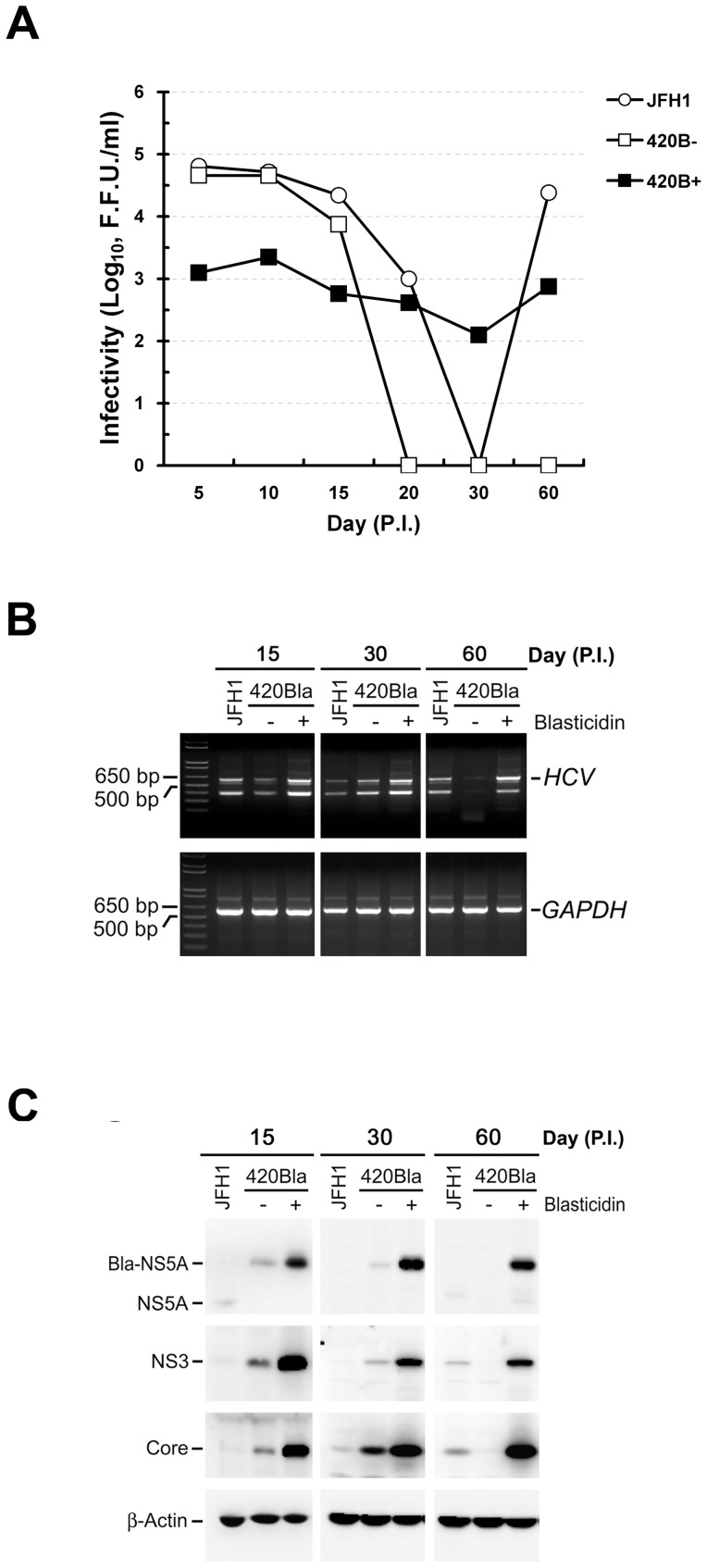
Characterization of long-term HCV-Bla infected stable cells. Huh7 cells were infected with JFH1 or 420Bla viruses at an MOI of 0.01 for 12 hr, and cells were cultured for additional two days. Half of the 420Bla virus-infected cells was cultured with or without blasticidin. At the indicated times, the extracellular viral infectivity (A), intracellular viral RNA amounts (B), and viral protein expressions (C) of infected cells were determined.

Furthermore, we examined whether constitutive and vivacious expressions of HCV RNA and proteins impede the virus propagation in infected cells. As shown for JFH1-infected cells, 420Bla-infected cells without selection robustly produced virus with a maximum infectivity of ∼10^5^ F.F.U./ml during 5 to 10 days post-infection and their viral infectivity fluctuated when cells entered the chronic infection phase (days 20 to 60) (Figure 5A). In contrast, the virus titer of 420Bla+ infected cells with selection was dramatically lower than that of 420Bla viral infection without selection at 5, 10, and 15 days after infection and fluctuated between 10^2^∼10^3^ F.F.U/ml at later culture times (Figure 5A). Nevertheless, viral RNA and proteins were more abundantly expressed in 420Bla+ infected cells with selection at 15, 30, and 60 days after infection, as compared to those obtained from non-selected ones as well as JFH1 infection (Figure 5B and C). These results reinforce that vigorous and sustained expressions of HCV RNA and proteins in long-term 420Bla+ stable cells do not necessarily warrant HCV propagation.

### HCV highly replicating cells are refractory to HCV reinfection

**Figure 6:**
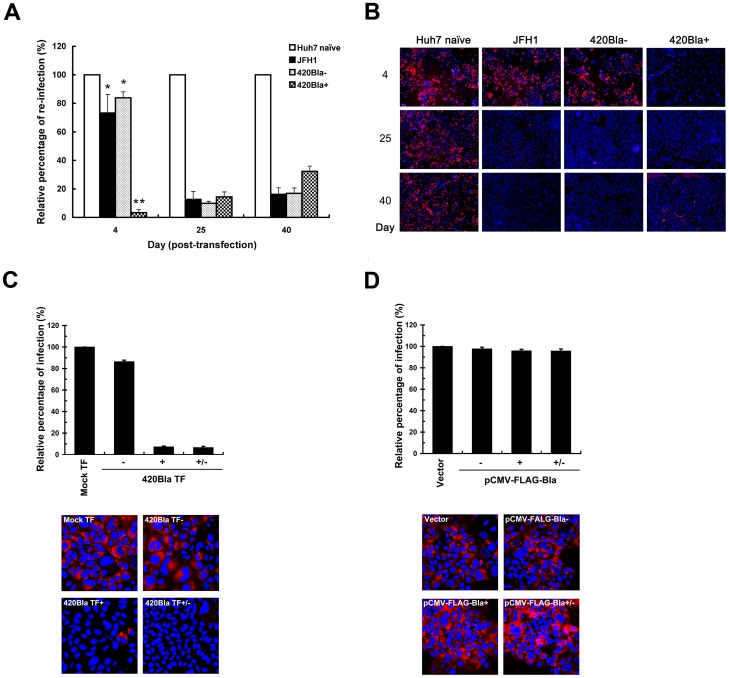
Interference with HCV reinfection in 420Bla+ stable cells as well as in chronically infected cells. (A and B) Huh7 cells were transfected with JFH1 or 420Bla RNAs, and cultured in the absence or presence of blasticidin. Huh7 naïve cells indicate the mock-transfected cells cultured in parallel. At the indicated times, Huh7 naïve, and JFH1-transfected 420Bla− and 420Bla+ cells were infected with 420RFP virus at an MOI of 0.05 for 12 hr and cultured for additional 3 days. Half of the 420RFP infected cell samples was quantified by flow cytometry (A) and immunofluorescence microscopy (B), respectively, for RFP-positive cells. The relative percentage of reinfection in viral RNA transfected cells was calculated by normalization with that of Huh7 naïve cells in (A). The Hoechst 33258 staining (blue) in (B) indicates the nuclei. (C) The Mock TF, 420Bla−, 420Bla+, and 420Bla+/− cells were established as described in Figure 3A, and the cells were analyzed for 420RFP reinfection as described above. (D) The Vector, FLAG-Bla−, FLAG-Bla+, and FLAG-Bla+/− cells from Figure 3E were also assayed for 420RFP virus reinfection as described above. Data represents mean ± SEM (n = 3).

We next investigated whether establishment of an HCV highly replicating state may interfere with infection with a second homologous virus at the early infection stage by comparing the reinfection efficiency of a 420RFP reporter virus (Figure 1B, scheme 4) among different cells by flow cytometry. The 420Bla+ cells at day 4 after RNA transfection strikingly lost their responsiveness to 420RFP reinfection, as opposed to JFH1 and 420Bla- cells, which still exhibited a considerably high degree of responsiveness to reinfection by 420RFP virus (Figure 6A). Additionally, all JFH1, 420Bla-, and 420Bla+ cells greatly lost their permissiveness to reinfection at days 25 and 40 post-transfection (Figure 6A). These observations were in accordance with the results from immunofluorescence analyses (Figure 6B). Moreover, removal of blasticidin from culture media of 420Bla+ stable cells for one day did not restore the permissiveness to 420RFP virus infection (Figure 6C). In contrast, the FLAG-Bla+ stable cells selected with blasticidin retained similar extent of permissiveness to 420RFP infection to those in vector-transfected and FLAG-Bla-transfected cells without drug selection (Figure 6D). These results collectively exclude the possibility that blasticidin unexpectedly interferes with the 420RFP reinfection in 420Bla+ stable cells.

**Figure 7:**
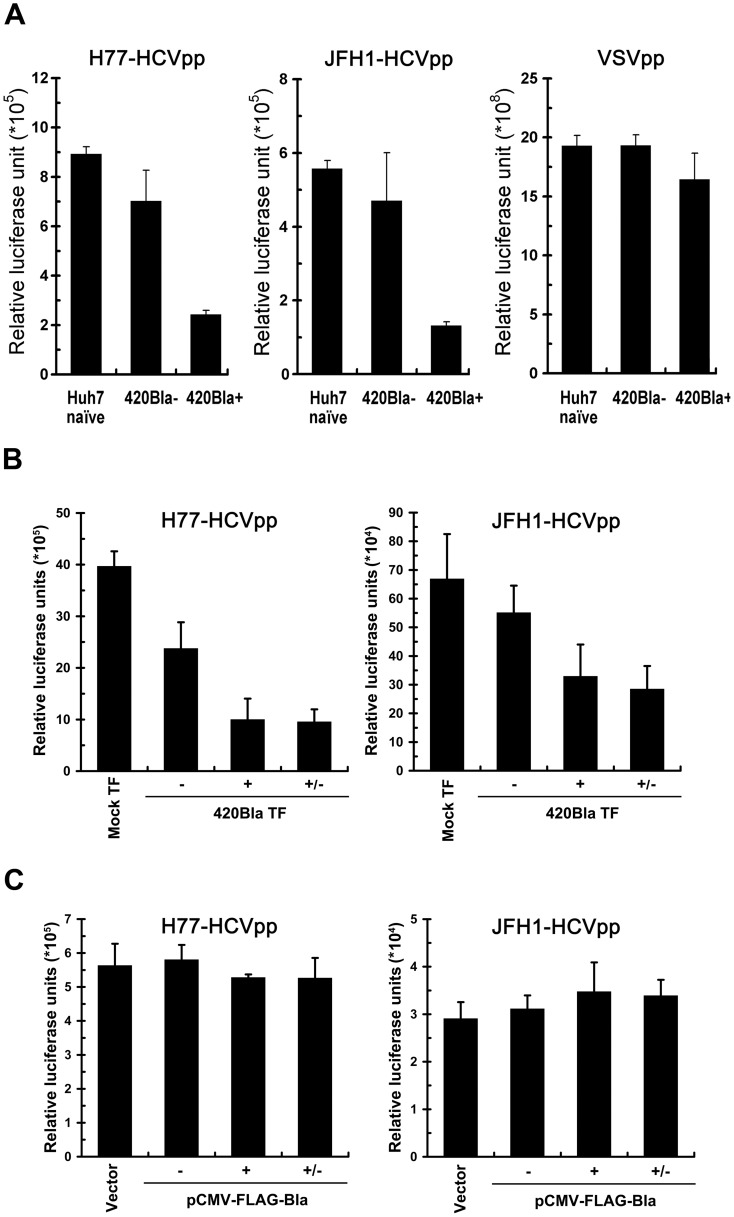
Inhibition of HCVpp infection in 420Bla+ stable cells. (A) Huh7 naïve, 420Bla−, and 420Bla+ stable cells selected with or without blasticidin for 4 days were infected with H77-HCVpp (genotype 1a), JFH1-HCVpp (genotype 2a), or VSVpp for 24 hr, and the firefly luciferase was measured 3 days post-infection. (B) The Mock TF, 420Bla−, 420Bla+, and 420Bla+/− cells from Figure 3A were also analyzed for H77-HCVpp (left panel) and JFH1-HCVpp (right panel) infection as described above. (C) Vector, FLAG-Bla−, FLAG-Bla+, and FLAG-Bla+/− cells from Figure 3E were assayed for H77-HCVpp (left panel) and JFH1-HCVpp (right panel) infection. Data represents mean ± SEM (n = 3).

Next, pseudotyped HCV particles (HCVpp) enveloped by HCV H77 (genotype 1a) and HCV JFH1 (genotype 2a) envelope glycoproteins E1 and E2 were employed to demonstrate the resistance to 420RFP reinfection of 420Bla+ stables, which was primarily resulted from a block to virus entry. In Figure 7A, the 420Bla+ stable cells greatly lost permissiveness to reinfection with H77- and JFH1-HCVpp but were still highly permissive to VSVpp infection as compared to Huh7 naïve and 420Bla− cells, indicating that the genotype–independent block to HCV reinfection in 420Bla+ cells is presumably from an inhibition of entry process of virus infection. Notably, the resistance to H77- and JFH1-HCVpp reinfection in 420Bla+ stable cells was not reversed by removal for one day of blasticidin from the culture media after selection (Figure 7B). In addition, no remarkable defect in H77- and JFH1-HCVpp infection in FLAG-Bla+ stable cells was detected (Figure 7C). These results argue again that highly active replication of HCV rather than blasticidin treatment renders 420Bla+ stable cells refractory to homologous virus infection.

### Active replication of HCV reduces the level of CD81 on the cell surface

**Figure 8:**
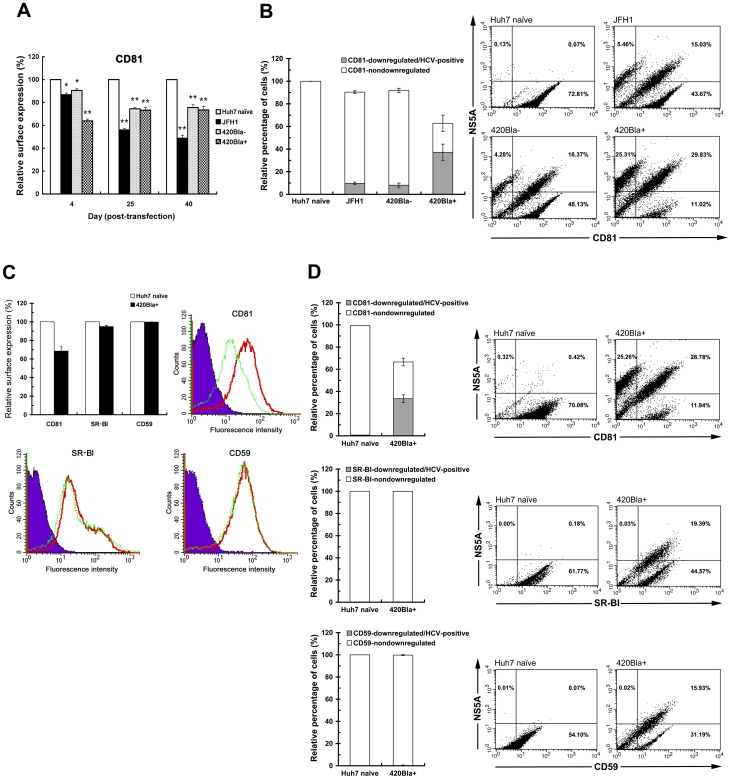
Downregulated CD81 surface level in 420Bla+ stable and chronically infected cells. (A) In parallel to the reinfection analysis of Figure 6A, the cell surface-expression of CD81 on Huh7 naive, JFH1, 420Bla−, and 420Bla+ cells was quantified by flow cytometry. The relative cell surface expression of CD81 at each time point was calculated by normalization with that of Huh7 naïve cells in the same set of samples. (B) Huh7, JFH1, 420Bla−, and 420Bla+ cells established as described in (A) were doubly stained for surface CD81 and HCV NS5A expression as described in “Materials and Methods”. A set of representative results from three independent experiments was shown in the right as two-dimension dot plots. The percentage of CD81-downregulated/HCV-positive cells was determined by calculating the ratio of cells located in the upper-left square to the total cells in each panel under different conditions. The percentage of “CD81-nondownregulated” cells was determined by calculating the ratio of the CD81 fluorescence-positive cells (upper-right and lower-right squares) to the total cells in each panel. In each calculation, the relative percentage of the ratio calculated under different conditions was quantified by normalization with that of Huh7 naïve cells. (C) Huh7 naïve and 420Bla+ stable cells as described above were analyzed for the cell surface expressions of CD81, CD59, and SR-BI. The histograms shown are a set of representative results from three separate CellQuest analyses. The purple shaded area, red solid line, and green dashed line indicate the IgG isotype control, Huh7 naïve cells, and 420Bla+ stable cells. (D) The Huh7 and 420Bla+ stable cells as described in (C) were doubly stained for the surface expression of indicated cell surface molecules and HCV NS5A as described in (B). The relative percentages of indicated surface molecule downregulated/HCV-positive and nondownregulated cells were quantified and calculated as described in (A). The two-dimension dot plots shown are the representative results from three independent experiments. Data represents mean ± SEM (n = 3). The statistical significances of the differences between the indicated and Huh7 naïve cells in (A) are determined by the two-tailed unpaired Student's *t*-test of the means (*P<0.05 and **P<0.01).

Since the exclusion to homologous infection in 402Bla+ highly replicating cells is primarily defective at the virus entry stage, we next examined whether interference with reinfection in 420Bla+ stable cells is resulted from reduction of the cell surface expression of CD81, a pivotal entry (co)receptor of HCV infection. The cell surface level of CD81 was decreased about 37% in 420Bla+ stable cells 4 days after transfection, as compared to that of Huh7 naïve cells (Figure 8A). In contrast, the cell surface expression of CD81 was slightly reduced in JFH1 RNA-transfected and 420Bla− cells at the same time point (Figure 8A). Long-term culture of JFH1-tranfected and 420Bla− cells for 25 and 40 days also led to notable reductions in the cell surface expression of CD81 (Figure 8A), which was in consistence with previous studies showing that serial passage of HCV in cells reduces the CD81 surface level [14]–[16].

We next analyzed the association of downregulated CD81 surface level with the HCV replication status in the 420Bla+ stable cells at 4 days posttransfection by double-labeling and flow cytometric assay. The gated cells harboring HCV expression and downregulated CD81 surface level were significantly increased in the 420Bla+ stable cells (∼25.31% of cells in Figure 8B), as opposed to those of JFH1 and 420Bla− cells (∼5.46% and 4.28% of cells, respectively, in Figure 8B), confirming that the reduction in the CD81 surface level in 420Bla+ stable cells was correlated with the highly replicating status of HCV. Meanwhile, the cell surface expressions of CD59, a glycosylphosphatidylinositol-anchored protein, and SR-BI, which also functions as an HCV entry (co)receptor, were not significantly reduced in 420Bla+ cells, as opposed to CD81 (Figure 8C). Despite the decrease of the CD81 surface level in 420Bla+ stable cells, there was no apparent downregulation of CD59 and SR-BI surface expressions in cells highly replicating HCV (Figure 8D). These results demonstrate that the decreased surface level of CD81 in 420Bla+ cells is not due to a general defect in presenting cell surface molecules onto plasma membrane (PM).

**Figure 9:**
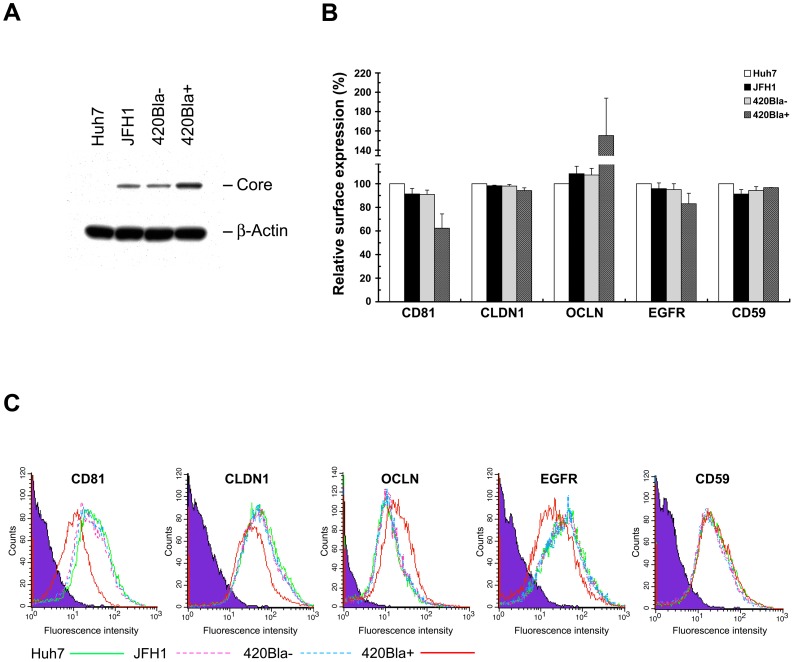
Analysis of the surface expressions of known HCV (co)receptors in 420Bla+ stable cells. (A and B) Huh7 cells were transfected with JFH1 or 420Bla RNAs, and cultured in the absence (−) or presence (+) of blasticidin for 3 days. Huh7 cells indicate the mock-transfected cells cultured in parallel. The cells were harvested for analyzing protein expressions (A) and surface expressions of HCV (co)receptors and CD59 by flow cytometry (B). (C) A set of representative results from three independent CellQuest flow cytometric analyses from (B) was shown as histograms. The purple shaded area, green solid line, pink dashed line, blue dashed line, and red solid line indicate the IgG isotype control, Huh7 cells, JFH1, 420Bla−, and 420Bla+ cells, respectively. Data represents mean ± SEM (n = 3).

To understand whether the surface levels of other known HCV entry (co)receptors are also downregulated by robust replication of HCV, we next compared the expressional changes of CLDN1, OCLN, and EGFR (co)receptors on the cell surface in JFH1, 420Bla−, and 420Bla+ cells at 4 days after RNA transfection. Consistently, the CD81 surface expression was greatly downregulated in 420Bla+ cells (Figure 9B and C). In contrast, no significant reduction of the CLDN1 and OCLN surface level was detected in 420Bla+ stable cells even the OCLN surface level seemed to be increased by stable expression of HCV (Figure 9B and C). Interestingly, the surface expression of EGFR was slightly reduced in 420Bla+ stable cells as compared to those of JFH1 and 420Bla− cells (Figure 9B and C), suggesting that cell surface EGFR may be another target regulated by active replication of HCV. These results collectively indicate that active replication of HCV leads to an impediment at viral entry via rapidly reducing the cell surface level of CD81.

### HCV stable replicon cells also reduce the CD81 level on the cell surface

**Figure 10:**
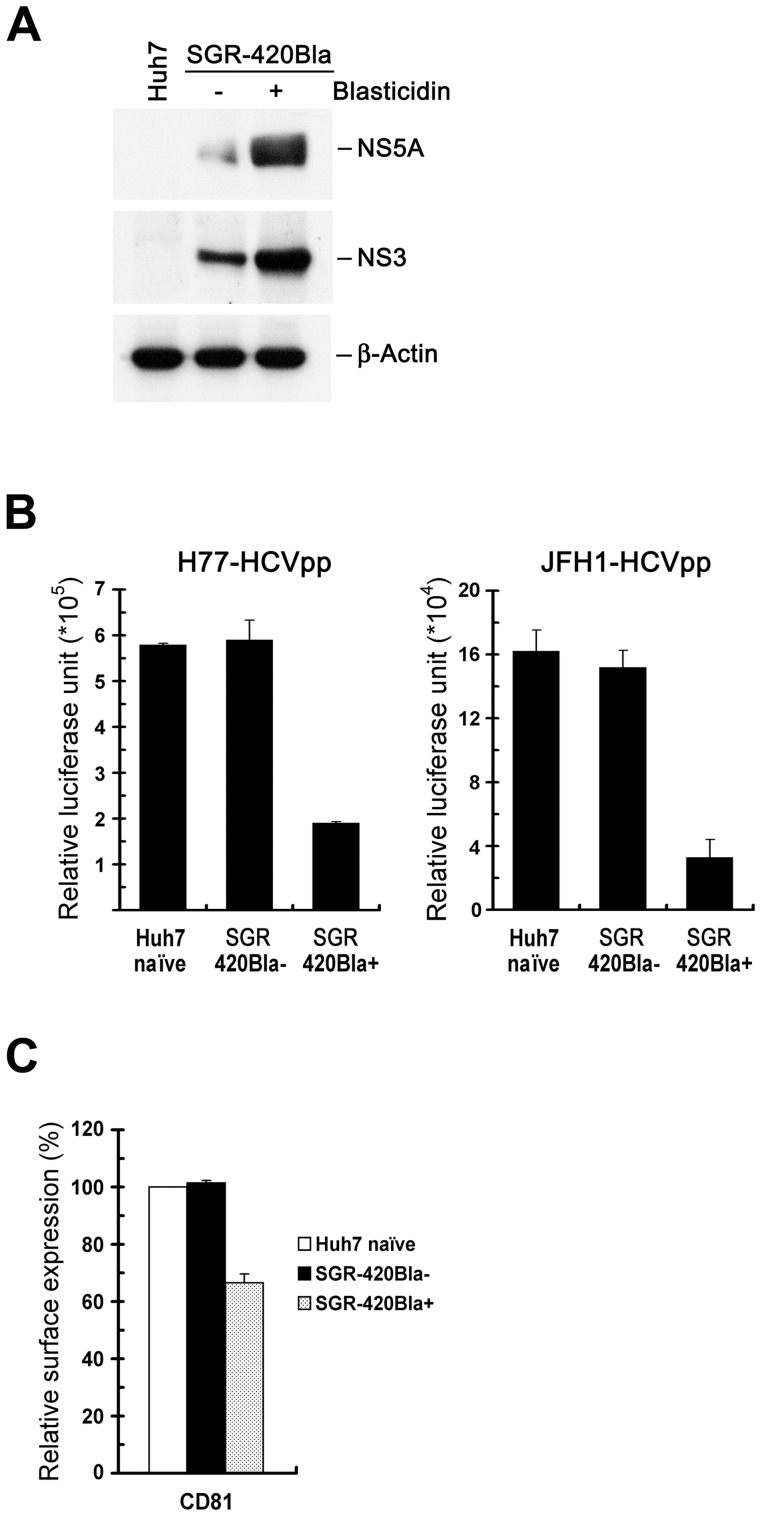
Downregulated cell surface expression of CD81 in SGR-420Bla+ stable cells. (A) Huh7 cells were transfected with SGR-420Bla RNA and one day after transfection, cells were selected with or without blasticidin for 10 days. The cells were analyzed for protein expressions of NS5A, NS3, and β-Actin. “Huh7” indicate the mock-transfected cells. (B) Huh7 naïve, SGR-420Bla−, and SGR-420Bla+ cells from (A) were infected with H77-HCVpp or JFH1-HCVpp for 24 hr, and the firefly luciferase was measured 3 days post-infection. (C) Huh7 naïve, SGR-420Bla−, and SGR-420Bla+ cells from (A) were determined for the cell surface expression of CD81 as described in Figure 8A. Data represents mean ± SEM (n = 3) (B and C).

To confirm that active HCV RNA replication indeed reduces CD81 expression on the cell surface, we employed a JFH1 SGR-420Bla replicon system that harbored a *Bla* gene at the 420 a.a. position of NS5A (Figure 1B, scheme 5). We found that Huh7 cells stably expressing SGR-420Bla were greatly resistant to HCVpp infection (Figure 10A and B). Also, the cell surface expression of CD81 was repressed in the SGR-420Bla+ stable cells (Figure 10C). These results suggest that active viral RNA replication, but not the complete viral life cycle, of HCV is sufficient to reduce the surface level of CD81, resulting in interference with HCV reinfection.

### Productive replication of HCV J6/JFH1 chimera and adaptive mutant of JFH1 rapidly induces downregulation of CD81 surface level

**Figure 11:**
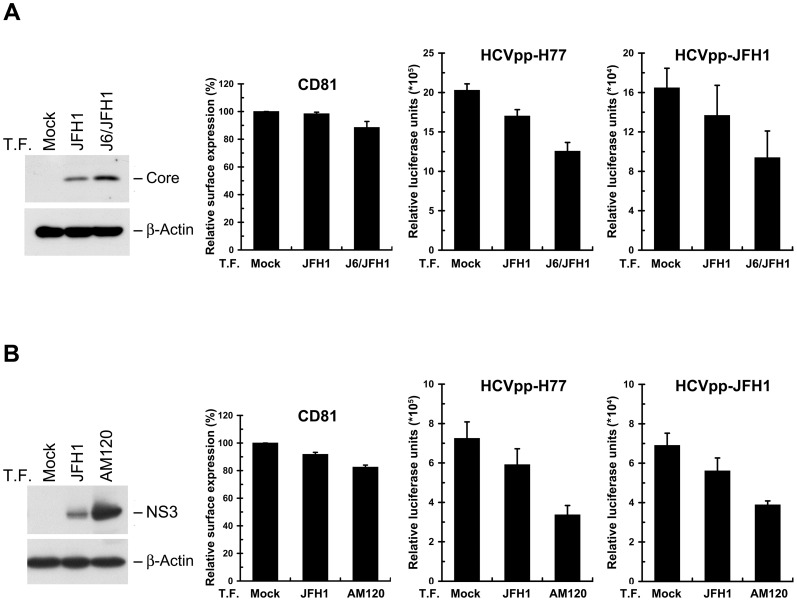
Promoted downregulation of CD81 surface level by enhancing HCV replication. Huh7 cells were mock transfected or transfected with JFH1 or J6/JFH1 viral RNA (A), or mock transfected or transfected with JFH1 or JFH1 AM120 adaptive mutant viral RNA (B). At day 3 posttransfection, the cells were analyzed for protein expressions (left panel), CD81 surface expression (middle panel), and H77-HCVpp and JFH1-HCVpp infection (right panels). Data represents mean ± SEM (n = 3).

To determine whether the surface level of CD81 and the sensitivity to HCV infection can be differentially controlled by high and low levels of HCV replication in cell cultures without the use of drug selection, we compared the downregulation of CD81 surface expression and the resistance to HCVpp infection between JFH1-tranfected cells and cells transfected with the J6/JFH1, a chimeric viral genome consisting of the structural genes (C, E1, and E2), p7, and NS2 from another genotype 2a strain (strain HC-J6) and the NS2 to 3′-UTR fragment of JFH1. This chimera was shown to produce a relatively higher titer of HCV than the parental JFH1 strain [11]. As shown in Figure 11A, transfection of cells with J6/JFH1 viral genome not only led to a higher degree of CD81 reduction on the cell surface but also rendered cells more refractory to HCVpp infection as compared to those of JFH1-transfected cells. Moreover, transfection of a JFH1 adaptive mutant, AM120, which was isolated from JFH1-transfected Huh7.5 cells serially passaged for 120 days and was shown to enhance virus production [30], also resulted in a higher extent of CD81 downregulation on the cell surface as well as increased resistance to HCVpp infection as opposed to those of wild-type JFH1-transfected cells (Figure 11B). These results conclude that the replication status of HCV decisively controls the expressional level of cell surface CD81, thus regulating the cell permissiveness to virus reinfection.

### Expression of CD81 is reduced and cytoplasmically retained in HCV highly-replicating cells

**Figure 12:**
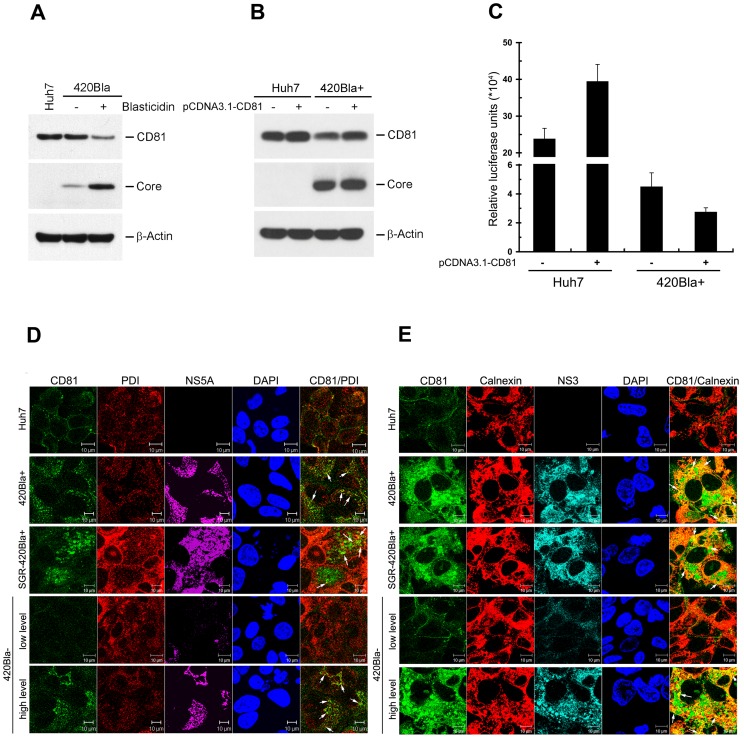
Impaired expression, cellular localization, and functional rescue of CD81 in HCV RNA highly replicating cells. (A) Huh7 cells were transfected with 420Bla and selected with or without blasticidin. Then cell lysates were analyzed for the total expressions of CD81, core, and β-Actin by Western blotting. (B and C) A portion of Huh7 and 420Bla+ stable cells established as described in (A) was transfected with pCDNA3.1-human CD81 plasmid DNA. Twenty-four hours after transfection, cells were analyzed for CD81 expression (B). In parallel, another portion of cells was infected with JFH1-HCVpp for 24 hr, and the firefly luciferase was measured at 72 hr post-infection (C). The “−” indicates mock-transfected cells. Data represents mean ± SEM (n = 4). (D–E) Huh7 cells were transfected with 420Bla or SGR-420Bla RNAs, and then selected with (+) or without (−) blasticidin. Four days later, cells were fixed and immunostained for the subcellular localization of CD81, PDI, Calnexin, NS5A, and NS3 as indicated. The white arrows indicate the colocalization signals of CD81 with PDI (D) or Calnexin (E) shown in each merged image. The PDI and Calnexin staining indicate the localization of ER. The staining of DAPI, NS5A (D) and NS3 (E) indicates the nuclei and HCV-expressing cells, respectively.

We then determined what causes the decreased CD81 level on the cell surface in 420Bla+ transfectants. Western blotting analysis showed that the total CD81 level in 420Bla+ stable cells was lower than that observed with 420Bla− cells without selection (Figure 12A). Nevertheless, real-time PCR quantification showed that the mRNA level of CD81 was not significantly changed in 420Bla+ stable cells as compared to Huh7 naïve and 420Bla− cells (data not shown). These observations suggest that the reduced CD81 level in 420Bla+ stable cells may occur through a post-translationally regulatory mechanism on CD81 in these stable cells. Furthermore, we investigated whether overexpression of CD81 can reverse a block in HCV reinfection in 420Bla+ stable cells (Figure 12B and C). As seen in Figure 12C, the established 420Bla+ stable cells still exhibited a 5-fold reduction in HCVpp entry, as compared to Huh7 naïve cells (compare columns 1 in the left and right panels of Figure 12C). In contrast to the results observed in Huh7 naïve cells, ectopic expression of CD81 in 420Bla+ stable cells did not restore the permissiveness to HCVpp infection (Figure 12C), which was consistent with the observation that the level of exogenously overexpressed CD81 in 420Bla+ cells was also lower than that observed in Huh7 cells overexpressing exogenous CD81 (Figure 12B). These results indicate that highly active viral RNA replication in 420Bla+ cells may confer a state dominantly reducing the total and cell surface CD81 expression even when CD81 is ectopically overexpressed.

We next examined whether the reduced CD81 level in HCV RNA highly replicating cells was accompanied with altered subcellular localization of CD81. As previously reported [33], [34], a major population of CD81 was localized on the PM and concentrated in the intercellular junctional regions in Huh7 cells (row 1 in Figure 12D and E). In contrast, CD81 disappeared from the intercellular borders and was accumulated as cytoplasmic dot-like patterns which were partially colocalized with endoplasmic reticulum-associated proteins PDI and Calnexin in 420Bla+ and SGR-420Bla+ cells (rows 2 and 3 in Figure 12D and E, respectively). Similarly, a fraction of PDI and Calnexin was also colocalized with the intracellular punctate structures of CD81 in the 420Bla− cells with a higher expression level of HCV, but not in those with a lower HCV RNA level (rows 5 and 4 in Figure 12D and E, respectively). These results conclude that active replication of HCV RNA leads to reduction of the total expression and sequestration of CD81 within the cytoplasm, thus decreasing its level on the cell surface.

## Discussion

In the present study, we employed a stable cell model to differentially express HCV and determine its subsequent effect on virus propagation. This system is capable of “synchronizing” and enriching a “quasi homogenous” cell population maintained at an HCV highly-replicating state, versus cells expressing normal levels of HCV. As originally thought for an HCV stably expressing clone, established 420Bla+ transfectants with blasticidin selection produced more abundance of viral RNAs and proteins as well as infectious virus, compared to JFH1 RNA-transfected and 420Bla− cells (Figure 2). Intriguingly, highly active viral RNA replication and maintenance of a high portion of HCV-expressing population in the long-term 420Bla+ stable cells failed to support sustained high-titered virus production (Figure 4), as was found in other HCV stable cell models previously reported [19], [20], [26]. As did the JFH1 virus, infection with 420Bla virus without selection produced high-tittered viral infectivity in the early stage of infection, which was followed by fluctuated infectivity in the latter phase of infection (Figure 5). Nevertheless, 420Bla virus infection with blasticidin selection persistently produced virus with a much lower infectivity than those produced without selection despite their expression of high levels of viral RNA and proteins (Figure 5). These observations were further supported by single clone analyses on transfected or infected 420Bla+ cells (Figure 4A and B). Collectively, these results explain the failure of producing high-titered virus in the previously established HCV stable cultures [19], [20], [26].

It is not unexpected that productive replication of HCV in 420Bla+ stable cells induces severe cytopathic effects, e.g., inhibition of cell growth (Figure 3A and B). This observation is consistent with the previous report showing retarded cell growth in HCVcc cell culture [16]. Particularly, the unfolded protein response and autophagy were activated in the HCV 420Bla+ cells (data not shown), suggesting that actively replicating HCV in cells can trigger a massive stress response to interfere with cell growth. Since the surface levels of CD59 and other HCV entry (co)receptors are not apparently downregulated and since the permissiveness to VSVpp infection is unaffected in the HCV 420Bla+ stable cells (Figure 7, 8, and 9), it is conceivable that the reduction of CD81 surface level and exclusion to HCV reinfection by actively replicating HCV is not resulted from a defect in cell growth. Moreover, CD81 has been shown to regulate cell growth in hematopoietic linage cells [35], [36], and play a critical role in the tumorigenesis as well as in the migration of tumor cells via regulating the mitogen activating signaling and cells motility [37], [38]. These findings collectively imply that downregulated CD81 by productive replication of HCV may act as a critical factor to control cell growth and modulate the pathogenesis of HCV-related liver diseases.

Unlike the naïve Huh7 cells and JFH1 RNA- and 420Bla-transfected cells without selection, which established superinfection exclusion at a later stage after genomic RNA transfection, the rapid emergence of refractoriness to HCV reinfection in 420Bla+ stable cells was coincidently associated with rapid reduction of the cell surface level as well as reduced total expression of CD81 (Figure 6, 7, 8 and 12A). Further replicon as well as J6/JFH1 and JFH1 AM120 adaptive mutant analyses showed that highly active HCV RNA replication, per se, is sufficient to interfere with HCV reinfection via decreasing the surface level of CD81 (Figure 10 and 11). It should also be noted that HCV RNA productively replicating cells did not contain any apoptotic cells and the total mRNA of CD81 was not significantly changed in 420Bla+ stable cells (data not shown). Moreover, 420Bla+ stable cells ectopically expressing CD81 were still refractory to HCVpp entry (Figure 12B and C), and CD81 was retained in the cytoplasm and colocalized with endoplasmic reticulum-associated proteins in HCV RNA highly replicating cells (Figure 12D). These results together indicate that productive viral RNA replication somehow triggers cellular machinery to downregulate CD81 expression through an undefined mechanism, resulting in the reduced CD81 cell surface expression. The study also indicates that the reduced surface level of CD81 is not the outcome of evolutionary selection of cells with low permissiveness for HCV infection, as were previously reported [15], [16].

CD81 has been shown to function as a pivotal role in both cell-free virus infection and cell-to-cell viral transmission [39], [40]. On the other hand, previous neutralization antibodies studies showed that the neutralized CD81 Ab partially inhibits the cell to cell transmission of HCV and this route of virus transmission is also dependent on other entry (co)receptors such as CLDN1 and SR-BI [39], [40]. Due to the unchanged surface levels of CLDN1 and SR-BI in 420Bla+ stable cells, (Figure 8C, 8D, 9B, 9C), it is of interest to further investigate whether the HCV replicating status could regulate the cell-to-cell transmission of HCV.

In the present study, we found that among known HCV entry (co)receptors CD81 is the only (co)receptor whose surface expression is dramatically downregulated in cells highly replicating HCV though the EGFR surface level was slightly decreased (Figure 8 and 9). Why and how only the surface level of CD81 is downregulated in HCV infection still remain elusive. HCV infection causing selection of a cell population expressing a low level of CD81 and endocytosis of CD81 along with the internalized viral particles have been implicated in downregulation of CD81 from the cell surface [15], [16], [18]. In the future, it is worthwhile to determine whether robust replication of HCV facilitates the internalization process of CD81 or whether productive replication of HCV may activate a cellular response to retain CD81 within endoplasmic reticulum-related compartments, thus inhibiting the presentation of CD81 onto the surface. For instance, active HCV replication may trigger a stress response to induce polyubiquitination of CD81, thereby targeting polyubiquitinated CD81 for degradation. In line with this hypothesis, Lineberry et al. has shown that CD81 could be polyubiquitinated and degraded by Lys-48 linkage through the E3 ubiquitin ligase activity of gene related to anergy in lymphocyte (GRAIL) [41]. Further analysis is needed to determine whether HCV replication can activate the GRAIL expression or enhance the E3 ligase activity of GRAIL to induce CD81 polyubiquitination and degradation.

Moreover, it is also interesting to investigate why the surface levels of other entry (co)receptors are not changed by productive HCV replication. In particular, SR-BI and CLDN1 have been shown to closely link to CD81 in the entry process of HCV virion. Although CLDN1 and OCLN were shown to be downregulated by HCV through envelope proteins-mediated cytoplasmic retention of these two (co)receptors [14], [17], it should be noted that in our study the HCV RNA replication-mediated (co)receptor downregulation mainly depends on the replication of viral genome since transfection of replicon RNA lacking the envelope genes can still trigger downregulation of CD81 (Figure 10). On the other hand, a recent study showed co-endocytosis of CLDN1 with CD81 via the HCV internalization process, implying that the CLDN1 surface expression may be reduced during the entry of HCV virion into target cells [18]. On the contrary, the CLDN1 level was shown to be upregulated in the HCVcc-infected hepatocyte and the liver tissue from HCV-infected individuals [34]. The regulation of CLDN1 expression by multiple viral and cellular factors may explain why the cell surface level of CLDN1 seems unchanged in the 420Bla+ cells.

So far, whether HCV can downregulate the SR-BI cell surface level has not been answered. Along this line, our study points out that the SR-BI surface level is not targeted or regulated by HCV. On the other hand, it has been shown that SR-BI expression is regulated by mitogen-activated protein kinase (MAPK) activity in hepatic cells [42]. Also, the expression of SR-BI can be transcriptionally regulated by sterol-regulatory-binding protein (SREBP) signaling [43]–[45]. Since HCV infection can activate MAPK and SREBP downstream signaling [3], [46], [47], it is likely that HCV replication may upregulate SR-BI expression in a transcriptional or posttranscriptional manner, thus masking the downregulation effect on the surface level of this (co)receptor. However, further investigation is necessary to discern these hypotheses.

Based on the results obtained here, we propose a model to illustrate how active viral RNA replication counteracts virus propagation. At the early stage of infection, active HCV RNA replication leads to productive formation of infectious virus and virus spread. Nevertheless, in response to robust HCV RNA replication, cells somehow trigger an unknown mechanism to downregulate the total CD81 expression and sequestrate CD81 within the ER-associated compartment, leading to impaired ongoing virus transmission and persistent infection. Also, the HCV replication status, which is influenced by the initial infecting virus load or the genotype of infecting HCV, can determine the rapidness of occurrence of viral persistence. In sum, our study not only reveals the importance of the CD81 cell surface level in viral persistence, but also demonstrates for the first time that HCV RNA replication acts as a crucial viral determinant in modulating the expression and intracellular localization of CD81, thus controlling viral propagation.
